# Total MRI burden of cerebral vessel disease correlates with the progression in patients with acute single small subcortical strokes

**DOI:** 10.1002/brb3.1173

**Published:** 2018-12-03

**Authors:** Jingwen Jiang, Xiaojun Huang, Yu Zhang, Weiping Deng, Fanxia Shen, Jianrong Liu

**Affiliations:** ^1^ Department of Neurology Ruijin Hospital North Affiliated to Shanghai Jiao Tong University School of Medicine Shanghai China

**Keywords:** cerebral small vessel disease, progression, single subcortical stroke, total MRI burden

## Abstract

**Background:**

The patients of single small subcortical strokes (SS) commonly have neurological worsening with risk factors, and mechanisms remain unclear. Asymptomatic lacunes, white matter lesions, cerebral microbleeds, and enlarged perivascular spaces are MRI markers of cerebral small vessel disease (cSVD). Previous studies mostly explored the association between the neurological deterioration and presence of above markers separately. The relationship between progressive single small SS and the simultaneous presence of multiple markers of cSVD has not been fully identified. We aimed to investigate whether total burden of cSVD detected with MRI was associated with progressive small SS in this study.

**Methods:**

Patients with single small SS (2.0 cm in diameter) were prospectively recruited during January 2016 and May 2018. Progression was defined as worsening by ≥1 point in National Institutes Health Stroke Scale (NIHSS) motor score within 72 hr from onset. The presence and burden of cSVD were determined by brain MRI, producing a score between 0 and 4. Besides, the patients' characteristics, clinical data, medical treatments during hospitalization stay were collected and statistically analyzed. Associations with progression were tested with forward stepwise regression analyses.

**Results:**

Fifty‐seven (35.6%) patients underwent progression. No significant difference was observed in the distribution of any single vascular risk factor and its related laboratory data among these patients. After adjustment for age, sex, NIHSS score at admission, and time from stroke to MRI in separate models, severe WMHs (OR = 4.892; 95% CI = 2.011–11.904, *p* = 0.016), moderate‐ and high‐grade basal ganglia EPVS (OR = 2.970; 95% CI = 1.861–6.121, *p* = 0.009), and total cSVD score (OR = 3.359; 95% CI = 2.016–5.599, *p* = 0.010) were associated with progression.

**Conclusion:**

This study demonstrated that total MRI cSVD burden was independently associated with progression after single small subcortical strokes.

## 1 INTRODUCTION

Single small subcortical strokes (SS), also known as lacunar infarcts, are clinically defined as a brain infarction <20 mm in diameter accompanied by lacunar syndrome as described by National Institute of Neurological Disorders and Stroke (1990). According to previous epidemiological surveys, single SS account for 20%–30% of all cerebrovascular infarctions (Kolominsky‐Rabas, Weber, Gefeller, Neundoerfer, & Heuschmann, 2001; Wolfe et al., 2002). As reported, about 12%–36% of the patients with SS underwent neurological worsening in hours or even days after stroke onset (Nakamura, Saku, Ibayashi, & Fujishima, 1999; Ohara, Yamamoto, Tamura, Ishii, & Murai, 2010; Yamamoto et al., 2010). The early neurological deterioration involving motor function is not rare even with acute medical treatment (Saia & Pantoni, 2009; Staaf, Geijer, Lindgren, & Norrving, 2004) and could frequently leave a serious disability. Although several clinical factors, including high hemoglobin Alc (HbAlc) levels (Isa et al., 2012), history of hypertension (Yamamoto, Bogousslavsky, & Melle, 1998), and enlargement of lesion size (Terasawa et al., 2008; Yamada et al., 2004), have been reported to be associated with neurological deterioration in SS patients, neither a specific predictor nor an exact mechanism has been identified as yet (Bene et al., 2012). There seems to be no suitable means of intervention could suppress neurologic progression or improve functional outcomes in SS patients (Takeuchi et al., 2016). It is of great importance to identify the factors being associated with progression during the early period, as well as exploring suitable personalized treatments to prevent clinical worsening.

Progression with single SS (2.0 cm in diameter) yet cannot be fully explained by branch atheromatous disease (BAD) when there was no evidence of stenosis in proximal segment or the orifice of the penetrating artery (Nakase, Yoshioka, Sasaki, & Suzuki, 2013). The presence of extensive white matter hyperintensities (WMHs) in lacunar stroke patients was associated with higher number of lacunar infarcts, which could support the hypothesis that both multiple small SS and WMHs may share the common pathogenesis: cerebral small vessel diseases (Csvd; Boiten, Lodder, & Kessels, 1993; Micheli & Corea, 2012; Oscar Benavente & Streifler, 2001). cSVD refers to various pathological processes that affect microvasculature in the brain, which is generally used to describe a series of neuroimaging changes in the white matter and subcortical gray matter, including white matter hyperintensities (WMHs), enlarged perivascular spaces (EPVS), silent lacunar infarctions (SLIs), and cerebral microbleeds (CMBs) in brain magnetic resonance imaging (Pantoni, 2010). As proved by previous studies, WMHs not only predict a higher incidence of future stroke (Smith, 2010), but also be independently associated with progression of small subcortical infarction (Feng et al., 2014). While MRI imaging markers of cSVD do not occur separately in a patient, a total cSVD burden might better represent the overall degree of cSVD; thus, a validated scale of the ordinal MRI imaging burden of cSVD has been proposed by counting the presence of each four MRI imaging features (Staals, Makin, Doubal, Dennis, & Wardlaw, 2014). The total cSVD scale has been identified to be correlated with chronic kidney disease (Jin et al., 2014) and cognitive function (Staals et al., 2015) in ischemic stroke patients, but the combined effect of these MRI imaging markers of cSVD on single small SS patients during subacute phase has never been studied. In this study, we aimed to investigate the clinical features and total burden of cSVD in patients with single small SS and explore their predictive value for neurological deterioration of SS during subacute phase.

## 2 SUBJECTS AND METHODS

### 2.1 Ethics

This study has received ethics approval from the Ruijin North Hospital (2016 Ruibeilunshen No (1)‐2).

### 2.2 Subjects

This was a hospital‐based, cross‐sectional observational study. Participants consisted of a sample of consecutive patients admitted to the Department of Neurology in the Ruijin North Hospital affiliated to Shanghai Jiao Tong University Medical School between January 2016 and May 2018 with a diagnosis of acute single small subcortical stroke. The inclusion criteria were as follows: (a) The patient should meet the clinical lacunar stroke syndrome (modified from the Fisher criteria; Miller Fisher, 1991) with focal neurologic deficit; (b) Acute single small subcortical strokes identified by MRI were located in deep regions, including the thalamus, gangliocapsular regions, corona radiate, and pons (Fisher, 1965) within 48 hr after stroke onset, with diffusion‐weighted imaging (DWI) lesion ≤2.0 cm in size at the largest dimension and corresponding to the clinical syndrome; (c) There is no evidence of >50% stenosis and non‐calcified plaques in large vessel (internal carotid, middle cerebral, or basilar intracranial artery) identified by magnetic resonance (MR) angiography (Micheli & Corea, 2012); (d) The patients with a possible cardioembolic source (most commonly atrial fibrillation or a valvular prosthesis) were excluded. Progression was defined as an increase in the National Institutes Health Stroke Scale (NIHSS; Kasner et al., 1999) motor score by 1 point or more during the first 72 hr after stroke onset (Kwon, Lim, Park, & Lee, 2011).

### 2.3 Collection of demographic and clinical data

We recorded the following demographic characteristics and vascular risk factors: age, sex, hypertension, diabetes mellitus, hyperlipidemia, coronary heart disease, history of smoking, and drinking (Yang et al., 2015). Neurological deficits based on the NIHSS scores were measured at admission and daily throughout the patient's stay at the hospital by one of the three designated neurologists. The measurement of the NIHSS scores in this study demonstrated excellent internal reliability with Cronbach's *α* value of 0.79.

### 2.4 MRI protocol and assessment

All patients had baseline CT scan upon arrival to the emergency room and underwent MRI scan with 24 hr from the stroke onset. MRI was performed on a 1.5T scanner or a 3.0T scanner with the following sequences obtained: axial T1‐weighted, axial T2‐weighted, fluid‐attenuated inversion recovery (FLAIR), diffusion‐weighted imaging, and axial susceptibility‐weighted imaging. Intracranial arteries were assessed by magnetic resonance angiography.

### 2.5 Cerebral small vessel disease burden scale

All the available scans (T1, T2, FLAIR sequences) were independently rated by two trained vascular neurologists for the presence and severity of cSVD features. In case of disagreement, a consensus meeting was held. We defined lacunes as round‐shaped cerebrospinal fluid isointense lesions measuring ≤20 mm in diameter on axial section in the white matter, basal ganglia, or brainstem on T1, T2, or FLAIR sequences (Wardlaw, Smith, Biessels, et al., 2013) and do not compatible with clinical findings. White matter hyperintensities (WMHs) were defined as focal or confluent hyperintensities in the deep and periventricular white matter on FLAIR images and graded using the modified Fazekas scale (Fazekas et al., 1993). Cerebral microbleeds (CMBs) were rated on susceptibility‐weighted imaging as homogeneous rounded lesions of signal loss, within a diameter <10 mm (Greenberg et al., 2009). Enlarged perivascular spaces (EPVS) were defined as ≤2 mm round or linear cerebrospinal fluid isointense lesions (T2 hyperintense and T1/FLAIR hypointense). We only counted EPVS at the level of the basal ganglia which were specifically identified to be associated with cSVD (Doubal, MacLullich, Ferguson, Dennis, & Wardlaw, 2010; Huijts et al., 2014; Zhu et al., 2010). An ordinal score ranging from 0 to 4 was constructed to reflect the total burden of cSVD. One point was awarded for each of the following items: moderate to extensive (10–25 or >25) EPVS in the basal ganglia (1 point if present); ≥1 asymptomatic lacune (1 point if present); periventricular WMH Fazekas score 3 or if deep WMH Fazekas score 2 or 3 (1 point if present); ≥1 deep CMBs (1 point if present; Huijts et al., 2013; Klarenbeek, Oostenbrugge, Rouhl, Knottnerus, & Staals, 2013). Limited intrarater reliability testing (50 scans) showed a good reliability with kappa values of 0.73 for the presence of EPVs, 0.82 for CMBs, 0.87 for WMHs, and 0.77 for LIs.

### 2.6 Statistical analysis

By the Kolmogorov–Smirnov test for normality test, data are presented as *n* (%) for categorical variables or as mean ± *SD* for parametric data. Categorical variables were compared using the chi‐square test, while the continuous variables were compared by the independent *t* test. We performed the forward stepwise logistic regression analysis and retain variables with *p *< 0.15 (as shown in Table 1) in multivariate models by adjusting for age, sex, NIHSS score at admission, and time from stroke to MRI. Finally, we analyzed association between the neurological deterioration and the presence of each MRI marker separately by performing logistic regression analyses. The statistically significant difference level was set at *p* < 0.05. All statistical analyses were conducted using SPSS version 24 (IBM Corp., Chicago, IL).

**Table 1:** Demographic and clinical characteristics of the study population stratified by the presence of progression

Variable	Patients without progression	Patients with progression	*p* Value
	*N* = 103	*N* = 57	
Age, mean years ± *SD*	62.23 ± 11.8	66.6 ± 11.5	0.140
Male, *n* (%)	56 (54.4)	28 (49.1)	0.526
Vascular risk factors, %			
Hypertension	71 (68.9)	41 (71.9)	0.705
Diabetes mellitus	24 (23.3)	19 (33.3)	0.121
Hyperlipidemia	20 (19.4)	10 (17.6)	0.355
Coronary heart disease	4 (3.9)	2 (3.5)	0.445
Current smoking	24 (23.3)	20 (35.1)	0.141
Drinking	22 (21.4)	10 (17.6)	0.614
Clinical data			
Thrombolytic treatment, %	9 (8.7)	3 (5.3)	0.256
Mono antiplatelets, %	70 (68.0)	37 (64.9)	0.695
Dual antiplatelets, %	33 (32.0)	20 (35.1)	0.493
Admission NIHSS Score	3.4 ± 3.3	4.6 ± 3.8	0.203
Discharge NIHSS Score	**2.0 ± 2.7**	**3.6 ± 3.0**	**0.012**
Time from stroke to MRI, hr	20.4 ± 7.3	20.1 ± 6.9	0.786
Laboratory data			
Triglyceride, mmol/L	1.74 ± 0.91	1.68 ± 0.81	0.893
Total cholesterol, mmol/L	4.75 ± 0.77	4.80 ± 0.98	0.868
Low‐density lipoprotein, mmol/L	3.14 ± 0.70	3.19 ± 0.61	0.694
HbA1c	6.02% ± 1.50%	6.62% ± 1.72%	0.138
Homocysteine, mmol/L	14.73 ± 7.05	15.39 ± 11.8	0.762

Note

Bold values are significant with *p* < 0.05.

HbA1c: high hemoglobin Alc.

## 3 RESULTS

Finally, among the 160 patients in the study, (mean age 65.14 ± 11.67 years), 51.8% of them were men. The mean hospitalization days were 13.8 ± 4.3 (range 2–25) days. Fifty‐seven (35.6%) patients showed neurological deterioration. The others made up the non‐progression group. Among the progressive group, 17 (29.8%) patients showed 1‐point worsening in NIHSS score, 25 (43.9%) patients experienced 2‐point worsening, 15 (26.3%) ones underwent an increase by 3 points, while none of them had >=4 points worsening in our study.

Demographic and clinical characteristics between patients are presented in Table 1. Among these patients, no significant difference was observed in the distribution of any single vascular risk factor or its related laboratory data. All the patients received antiplatelet treatment. 66.9% of patients were given mono antiplatelet treatment (aspirin or clopidogrel), while 33.1% of which took dual antiplatelets. No matter what kind of antiplatelet treatment the patients received, it was not significantly more frequent in the patients without progression compared to the progressive group in our study. Twelve patients were treated with intravenous tissue plasminogen activator, and three of them showed neurological progression during or after the treatment of rtPA. As expected, the mean NIHSS score at admission did not differ between the two groups (3.4 ± 3.3 vs. 4.6 ± 3.8, *p* = 0.203), and NIHSS score at discharge was significantly higher in the progressive group (2.0 ± 2.7 vs. 3.6 ± 3.0, *p* = 0.012).

The radiologic characteristics including total burden score of cSVD are listed in Table 2. Eighty‐one (50.6%) of patients had more than one lacunes. Seventy‐three (45.6%) patients showed irregular periventricular hyperintensities extending into the deep white matter (Fazekas score 3) or confluent deep WMLs (Fazekas score 2 or 3), and 50 (31.3%) patients had evidence of more than one CMBs. Patients with progression had more frequency of extensive WMHs and presence of CMBs compared to those who did not progress (as shown in Table 2, all *p* < 0.05). Moderate‐ and high‐grade BG‐EPVS (10–25 or >25) were more common in patients with progression (22 patients, 38.6%) than in those without progression (13 patients, 12.6%). Patients of the progressive group had higher periventricular, deep white matter, and total scores of WMHs according to the Fazekas scale than the other group (all *p* < 0.001). For cSVD burden, a total of 27 (16.9%) patients had a cSVD score of 0, showing no signs of white matter changes, lacunes, CMBs, or EPVS, and 22 (13.8%) patients presented with a cSVD score of 4. What's more, the progressive group showed relatively high grade of cSVD (*p* < 0.001).

**Table 2:** Neuroimaging features of the study population stratified by progression

Variable	Patients without progression	Patients with progression	*p* Value
	*N* = 103	*N* = 57	
Presence of lacunar infarcts	47 (45.6)	34 (59.6)	0.219
Scores of WMHs (Fazekas scale)			
Periventricular score	1.53 ± 0.72	2.13 ± 0.71	**<0.001**
Deep white matter score	1.43 ± 0.68	2.03 ± 0.74	**<0.001**
Total score	2.96 ± 1.35	4.16 ± 1.39	**<0.001**
Extensive WMHs^a^	36 (34.9)	37 (64.9)	**<0.001**
Presence of CMBs (≥1)	24 (23.3)	26 (45.6)	**0.027**
Moderate‐ and high‐grade BG‐EPVS (10–25 or >25)	13 (12.6)	22 (38.6)	**0.012**
Total cSVD Score *n* (%)			
0 point	25 (24.3)	2 (3.5)	**<0.001**
1 point	37 (35.9)	3 (5.3)	
2 point	20 (19.4)	15 (26.3)	
3 point	17 (16.5)	17 (29.8)	
4 point	4 (3.9)	20 (35.1)	

Note

Bold values are significant with *p* < 0.05.

BG‐EPVS: basal ganglia enlarged perivascular spaces; CMBs: cerebral microbleeds; WMH: white matter hyperintensities.

a Extensive WMHs: periventricular WMH (Fazekas 3) or deep WMH (Fazekas 2 + 3).

Four separate multiple logistic regression models were constructed to identify the independent predictive value of history of smoking, diabetes, HAlbc, and each marker of cSVD (extensive WMHs, CMBs, moderate and high grade of BG‐EPVS, and total burden score were added into 4 separate models) for neurological deterioration. The results showed that extensive WMHs, moderate‐ and high‐grade BG‐EPVS, and total cSVD score were associated with the presence of progression independent of age, sex, NIHSS scores at admission, and time from stroke to MRI. Among them, the association between moderate‐ and high‐grade BG‐EPVS and neurological deterioration was the strongest. The details of four logistic models are listed in Table 3.

**Table 3:** Adjusted associations between radiological features of small vessel disease and stroke progression

Variable	Adjusted OR (95%)	*p* Value
Model 1		
Current smoking	2.349 (0.809,6.897)	0.120
Diabetes mellitus	0.422 (0.095,1.881)	0.258
HAlbc	1.198 (0.822,1.748)	0.347
Extensive WMHs	**4.892 (2.011,11.904)**	**0.016**
Model 2		
Current smoking	2.134 (0.702,5.932)	0.241
Diabetes mellitus	0.474 (0.120,1.889)	0.204
HAlbc	1.073 (0.784,1.532)	0.487
Two or more CMBs	1.471 (0.703–2.452)	0.263
Model 3		
Current smoking	2.463 (0.902,6.543)	0.314
Diabetes mellitus	0.609 (0.163,1.925)	0.320
HAlbc	1.278 (0.912,1.853)	0.217
Moderate‐ and high‐grade BG‐EPVS	**2.970 (1.861–6.121)**	**0.009**
Model 4		
Current smoking	2.093 (0.609,4.986)	0.374
Diabetes mellitus	0.563 (0.152,1.891)	0.392
HAlbc	1.063 (0.612,1.392)	0.502
Total cSVD Score	**3.359 (2.016–5.599)**	**0.010**

Note

Bold values are significant with *p* < 0.05 by adjusting for age, sex, NIHSS score at admission, and time from stroke to MRI.

BG‐EPVS: basal ganglia enlarged perivascular spaces; CI: confidence interval; CMBs: cerebral microbleeds; cSVD: cerebral small vessel diseases; OR: odds ratio; WMHs: White matter hyperintensities.

## 4 DISCUSSION

In our study, we excluded SS patients with BAD or with diagnosed cardioembolic sources. 35.6% of patients with single subcortical stroke showed progression in the first 72 hr after stroke onset. No significant difference was observed in the distribution of any single vascular risk factor or its related laboratory data between these two groups. The results of the present study indicated that severe preexisting WMHs, moderate‐ and high‐grade basal ganglia EPVS, and total burden of cSVD quantified with a combined score were associated with increased odds of progression in subcortical stroke patients after adjustment for age, sex, NIHSS score at admission, and time from stroke to MRI. Presence of lacunes and CMBs showed no association with clinical outcomes of single small SS patients in this study. Thus, it supplied new evidence for the close association between the total burden of cSVD and progressive single subcortical stroke.

In accordance with previous studies in SS patients, the study showed about one‐third of subcortical stroke patients having neurological deterioration (Audebert, Pellkofer, Wimmer, & Haberl, 2004; Nakamura et al., 1999). A number of studies have been conducted to investigate the potential predictors of progression in stroke patients. Kwon, Lee, Bae, and Kang (2014) reported the triglyceride level, total cholesterol, and homocysteine level were associated with deterioration in acute stroke patients. Yamamoto et al. (1998) observed that history of hypertension and age <64 years were independently associated with neurological deterioration among the stroke patients with small artery diseases. In other clinical studies, progression in lacunar stroke patients has been associated with older age and higher blood pressure values on admission (Kitanaka & Teraoka, 1995). Lesion location (internal capsule (Donnan, O'Malley, Quang, Hurley, & Bladin, 1993), pons (Saposnik, Noel de Tilly, & Caplan, 2008), and corona radiate (Nagakane, Naritomi, Oe, Nagatsuka, & Yamawaki, 2008)) and severity of motor deficit at admission (Yamamoto et al., 1998) were also consistently reported as the significant predictors of progression. Most of the above risk factors which were associated with atherosclerosis were reported as early predictors, corroborating the hypothesis that BAD was the main cause of progression in SS patients (Nakase et al., 2013). Our study excluded the patients with atherosclerosis of the parent artery or occlusion by proximal emboli, and there was no significant association between the presence of vascular risk factors and clinical progression of subcortical stroke. Wardlaw et al. (2011) also found no evidence at all of association between lacunar strokes and intracranial stenosis, supporting the theory that BAD and lacunar strokes may not share common pathogenesis. The single small SS (lacunar strokes) is not always a benign disease, and the studies with long‐term follow‐up were reported up to 25% of patients have a second stroke within 5 years (Samuelsson, Soderfeldt, & Olsson, 1996). Sacco et al. (2006) found an annual mortality rate of 13.0% and an annual recurrence rate of 2.8% in a population with lacunar stroke. Therefore, there might be other risk factors of an adverse outcome for small subcortical infarctions without BAD (Micheli & Corea, 2012).

This study demonstrated that extensive WMHs had an adverse impact on course of disease in acute small SS patients. Nannoni et al. (2015) identified that the presence of severe WMHs was independently associated with early neurological worsening in a study of 94 minor subcortical stroke patients, but they did not exclude the patient with BAD. Our study confirmed the close association between WMHs and progressive single small SS patients without BAD. Although there existed the differences in single small SS patient samples and definition of inclusion criteria, Feng et al. (2014) had the similar result in their study. As a common neuroimaging marker of cSVD, the mechanism of WMHs is identified to be associated with hypertensive hyaline degeneration, demyelination, and amyloid angiography in pathogenesis (Xiong & Mok, 2011), partly similar to small subcortical infarction which is thought to be caused by intrinsic vasculopathy of small perforators (Moran, Phan, & Srikanth, 2012). WMHs also have been found have an association with infarct volume and may lead to a larger growth of the infarct size during the hyperacute phase of ischemic stroke (Ay et al., 2008; Henninger et al., 2013). It has been widely accepted that vascular changes and ischemia generate WMHs (Auriel et al., 2011). Reduced cerebral blood flow and vascular reserve have been shown to be reduced in regions affected by WMHs, which is expected to reduce tissue survival in acute stroke (O'Sullivan et al., 2002). Both endothelial dysfunction and blood–brain barrier leakage are associated with WMHs (Wardlaw et al., 2009); the brain tissue is more likely to have impaired energy metabolism and self‐repair (Wang et al., 2012), which may lead to neurological worsening after stroke.

Perivascular spaces are fluid‐filled spaces surrounding the penetrating small vessels. EPVS in basal ganglia are considered as markers of hyperintensive small vessel disease (Zhu et al., 2010). Impaired perivascular drainage could further exacerbate solute deposition, including damaged peptides (e.g., leptomeningeal and superficial cortical vascular amyloid‐β), creating a feed‐forward loop (Martinez‐Ramirez et al., 2013). We also found EPVS in basal ganglia was independently associated with progressive‐type single subcortical stroke. Previous study showed EPVS in the basal ganglia was associated with disability (Arba et al., 2017), cognitive decline (Arba et al., 2018), and recurrence (Lau et al., 2017) in stroke patients, as well as depression (Sloten et al., 2015), sleep disorder (Berezuk et al., 2015), or movement disorders (Kim, Oh, & Kim, 2007) in stroke‐free populations. Thus, EPVS in the basal ganglia was a more valuable predictor for neurological deterioration than other neuroimaging markers of cSVD in our study, suggesting EPVS may be part of the pathologic spectrum that links to progressive SS.

According to a recent review on neuroimaging insights of cSVD, approaches to evaluate the total cSVD load on imaging are needed (Wardlaw, Smith, & Dichgans, 2013), and we used an established validated scale to grade the MRI burden of cSVD in this study. Considerably, we found signs of two or more severe cSVD features (total cSVD score ≥2) occupied 58.1% of our study population. Huijts et al. (2013) first applied the cSVD scale and found that accumulation of MRI burden in cerebral small vessel disease was accompanied by worse cognitive functioning. Elevated PCT levels (Li et al., 2018) and higher ambulatory blood pressure levels (Klarenbeek et al., 2013) were also identified to be positively associated with total burden of cSVD. Further studies on cSVD are now also needed to take into account the total cSVD load, avoiding heavily relying on just one feature as previous studies.

The strengths of our study are that we collected a homogeneous cohort of patients with single small subcortical (small vessel) stroke without BAD or cardioembolic stroke. What's more, we rated and qualitatified the extent of cSVD features by using validated scales which could be easily transferable to clinical practice. The small sample size and lacking long‐term follow‐up were the main limitations of our study. A larger data set is needed to better understand both mechanisms and factors underlying neurological worsening. The lesion location of the patients seems somehow to be neglected despite its unfavorable functional outcome. Besides lacking non‐invasive advanced high‐resolution MR angiography techniques, there existed difficulties in totally excluding the patients with BAD. Further studies including more samples and advanced methods are needed to validate our findings.

## 5 CONCLUSIONS

In accordance with previous studies in SS patients, the study showed about one‐third of subcortical stroke patients having neurological deterioration. Our study identified the predictive value of extensive WMHs, moderate and high‐grade BG‐EPVS, and total burden of cSVD for neurological progression after single small subcortical strokes. It suggested that the total burden of cSVD should be graded after acute single subcortical stroke for the evaluation of clinical course. Early rehabilitation might be appropriate for patients. We suggest further research into the clinical consequences of cSVD, including more samples and advanced methods with long‐term follow‐up, is needed to validate our findings.

## CONFLICT OF INTEREST

No potential conflict of interest was reported by the author(s).
